# Identification of large variation in *pfcrt*, *pfmdr*-1 and *pfubp*-1 markers in *Plasmodium falciparum* isolates from Ethiopia and Tanzania

**DOI:** 10.1186/s12936-015-0783-3

**Published:** 2015-07-08

**Authors:** Lemu Golassa, Erasmus Kamugisha, Deus S Ishengoma, Vito Baraka, Alex Shayo, Frederick N Baliraine, Nizar Enweji, Berhanu Erko, Abraham Aseffa, Angel Choy, Göte Swedberg

**Affiliations:** Aklilu Lemma Institute of Pathobiology, Addis Ababa University, Addis Ababa, Ethiopia; Department of Biochemistry, Catholic University of Health and Allied Sciences-Bugando, Mwanza, Tanzania; National Institute for Medical Research, Tanga, Tanzania; The Nelson Mandela African Institution of Science and Technology, Arusha, Tanzania; Department of Biology, LeTourneau University, Longview, TX USA; Armauer Hansen Research Institute, Addis Ababa, Ethiopia; Department of Medical Biochemistry and Microbiology, Uppsala University, Uppsala, Sweden; International Health Unit, Department of Epidemiology, University of Antwerp, Antwerp, Belgium

**Keywords:** Malaria, *Plasmodium falciparum*, *pfcrt*, *pfmdr*-1, *pfubp*-1, Ethiopia, Tanzania

## Abstract

**Background:**

*Plasmodium falciparum* resistance to anti-malarials is a major drawback in effective malaria control and elimination globally. Artemisinin-combination therapy (ACT) is currently the key first-line treatment for uncomplicated falciparum malaria. *Plasmodium falciparum* genetic signatures at *pfmdr*-1, *pfcrt*, and *pfubp*-1 loci are known to modulate in vivo and in vitro parasite response to ACT. The objective of this study was to assess the distribution of these resistance gene markers in isolates collected from different malaria transmission intensity in Ethiopia and Tanzania.

**Methods:**

*Plasmodium falciparum* clinical isolates were collected from different regions of Ethiopia and Tanzania. Genetic polymorphisms in the genes *pfcrt*, *pfmdr*-1 and *pfubp*-1 were analysed by PCR and sequencing. Frequencies of the different alleles in the three genes were compared within and between regions, and between the two countries.

**Results:**

The majority of the isolates from Ethiopia were mutant for the *pfcrt* 76 and wild-type for *pfmdr*-1 86. In contrast, the majority of the Tanzanian samples were wild-type for both *pfcrt* and *pfmdr*-1 loci. Analysis of a variable linker region in *pfmdr*-1 showed substantial variation in isolates from Tanzania as compared to Ethiopian isolates that had minimal variation. Direct sequencing of the *pfubp*-1 region showed that 92.8% (26/28) of the Ethiopian isolates had identical genome sequence with the wild type reference *P. falciparum* strain 3D7. Of 42 isolates from Tanzania, only 13 (30.9%) had identical genome sequences with 3D7. In the Tanzanian samples, 10 variant haplotypes were identified.

**Conclusion:**

The majority of Ethiopian isolates carried the main marker for chloroquine (CQ) resistance, while the majority of the samples from Tanzania carried markers for CQ susceptibility. Polymorphic genes showed substantially more variation in Tanzanian isolates. The low variability in the polymorphic region of *pfmdr*-*1* in Ethiopia may be a consequence of low transmission intensity as compared to high transmission intensity and large variations in Tanzania.

## Background

*Plasmodium falciparum* resistance to anti-malarials is a major drawback in effective malaria control in sub-Saharan Africa. Extensive use of chloroquine (CQ) as a monotherapy led to significant increase in levels of resistance across many malaria-endemic countries prompting policy changes. Post-CQ and sulfadoxine-pyrimethamine (SP) era, artemisinin combination therapy (ACT) such as artemether-lumefantrine (AL) has become the most important drug for the treatment of uncomplicated falciparum malaria in many endemic countries [1]. CQ resistance in *P. falciparum* malaria has been associated with *pfcrt* 76T (chloroquine resistance transporter gene) and *pfmdr*-1 86Y (multidrug resistance gene 1) alleles. Artemisinin partner drugs have been documented to exert opposing selective pressure on *pfcrt* and *pfmdr*-1 loci in previous studies [2–4]. Mutations in *pfmdr*-1 play a significant role in the parasite’s resistance to a variety of anti-malarials including lumefantrine [5, 6]. Indeed, the selection of *pfmdr*-1 86N by AL has been shown in a number of studies [7–10]. Likewise, a selection of *pfcrt* K76 was observed in a similar study [3]. Moreover, Djimde et al. [11] showed equal selection of *pfcrt* 76T and *pfmdr*-1 86Y alleles following artesunate plus amodiaquine treatment.

Understanding of the evolution of drug target genes under changing drug policy is crucial for drug efficacy monitoring using molecular markers [12]. Studies have shown that withdrawal of CQ was followed by the expansion of CQ-sensitive parasites possessing a wild type *pfcrt* allele in many countries [8, 13, 14]. The decline in CQ resistance after cassation of its use in many endemic areas suggests a possible re-introduction of CQ in the future [15] for chemoprevention or routine treatment. Such a decision requires close regional monitoring because evolution of CQ resistance after the removal of drug pressure seems to differ considerably among parasite populations. The fitness deficits incurred by mutant *pfcrt* K76T and *pfmdr*-1 N86Y strains are different. The K76T mutants are more strongly affected by the presence or absence of drug pressure than the *pfmdr*-*1* N86Y mutants. Haplotype “NFD” (N86, 184 F, and D1246) is associated with decreased susceptibility to AL and treatment with AL selects for this haplotype [16, 17]. The decline in drug resistance after removal of drug pressure could provide a new paradigm for anti-malarial treatment policies in Africa and may suggest a possible rotation of CQ in the future [18].

The reduction of malaria-related morbidity and mortality across sub-Saharan Africa gained in recent years as a result of the deployment of ACT, improved vector control and other measures could be compromised unless sustained ACT efficacy is maintained [19, 20]. Thus, the emergence of *P. falciparum* resistance to artemisinin derivatives in Greater Mekong sub-region jeopardizes these successes [21–23]. A few single nucleotide polymorphisms (SNPs) in one or two genes were shown to be responsible for resistance to CQ and SP [24]. These SNPs were then used as molecular markers to trace retrospectively the path of the resistant parasites. History has shown that for both CQ and SP, each resistant parasite population was introduced into East Africa from Southeast Asia (SEA) by individuals carrying the resistant strain, and then spread throughout the continent [25].

Unfortunately, artemisinin resistant phenotype, typified by delayed parasites clearance rates in the first few days after ACT treatment, was documented in 2009 in many patients from Cambodia [26], the same area where CQ and SP resistance started. Mutations in the *kelch* 13 gene have been associated with the slow clearance phenotype in Cambodian parasites [27]. This undesirable phenotype is steadily widening its territory and has spread across the Great Mekong sub-region [23, 28]. The new areas of slow parasite clearance would reflect a spread from a focal source just analogous to the “old” anti-malarials (CQ and SP) although an independent origin of artemisinin resistance has also been noted [29]. However, the variability of the K13 gene is low in African isolates and background mutations are distinct from the Asian genotypes [30].

The deubiquitinating enzyme gene (*pfcubp*-1) was first identified as a contributor to both CQ and artemisinin resistance in *Plasmodium chabaudi*. Mutations V739F and V770F were first identified to confer resistance to CQ and artesunate in rodents [31]. Later, *pcubp*-1 and *pcap2mu* (encoding clathrin vesicle-associated adaptor 2, µ subunit) genes were found to confer artemisinin resistance in *P. chabaudi* [32]. Both these genes are known to have polymorphic homologues in *P. falciparum*. A variant, E1528D, of the corresponding *pfubp*-1 was recently reported to be associated with reduced susceptibility to artemisinin in vitro [33]. Moreover, mutations in both the *pfubp*-1 and the *pfap2mu* genes have been contributing to the genetic signature of persisting sub-microscopic *P. falciparum* in ACT-treated Kenyan children [34]. Signs of recent selection of *pfubp*-1 related to use of artemisinin were noted [33] and polymorphisms in this gene may be shared determinants associated with slow clearance after artesunate monotherapy in Cambodia and Kenya [35]. Studies have shown that markers of resistance to CQ and amodiaquine (*pfmdr*-1) are known to be selected by ACT toward CQ-sensitive alleles [3, 36] implicated in artemisinin resistance. *Plasmodium falciparum* genome exhibits sequence variation that contributes to the pathogenic mechanisms of the parasite and determining the prevalence of resistance markers could provide a prediction about drug efficacy [37].

Malaria transmission is heterogenous in Ethiopia, ranging from highly seasonal and unstable areas to perennial transmission settings. The two major *Plasmodium* species causing malaria in Ethiopia are *P. falciparum* (about 60% of cases) and *P. vivax* (about 40% of cases) [38]. Ethiopia replaced CQ with SP as first-line drug for treatment of uncomplicated falciparum malaria in 1999, which in turn was replaced by AL in 2004, but CQ continues to be used for the treatment of *P. vivax* malaria.

In Tanzania, SP replaced CQ as a first line treatment of uncomplicated malaria in August 2001 which in turn was replaced by AL as a national first line treatment of uncomplicated malaria in 2006 [39]. But CQ is only used under restricted conditions for treatment of malaria in sickle cell disease patients. Despite the policy change in Tanzania, CQ continued to be available for prophylaxis of malaria in sickle cell disease patients. Scanty data are available on the effect of ACTs on the selection of molecular markers associated with the partner drug of the artemisinin derivative in areas with varying malaria transmission and therefore different level of drug pressure on the parasite population. The purpose of this study was, therefore, to determine polymorphisms in *pfubp*-1, *pfcrt* and *pfmdr*-1 genes in Ethiopia and Tanzania with AL as the first-line drug for uncomplicated falciparum malaria but where Ethiopia retained CQ for the treatment of *P. vivax* malaria.

## Methods

### Study population and site

In Ethiopia, the study was conducted in three sites (East Shoa, Gambella and West Arsi) from October 2012 through December 2014. East Shoa Zone is located 99 km from Addis Ababa, Ethiopia. Adama is the administrative town for the East Shoa Zone. The zone is found in the Great Rift Valley depression and malaria transmission is seasonal and unstable. Malaria transmission usually starts from September and ends in December. As it is true in most parts of Ethiopia, *P. falciparum* and *P. vivax* co-exist in the study area and malaria transmission is usually seasonal and unstable. In many malaria endemic areas of Ethiopia, the number of malaria cases substantially decreases (predominantly falciparum malaria cases) towards the end of December although vivax cases due to relapse appear in all months of the year. From East Shoa Zone, samples were collected from Adama malaria control centre. Gambella Town is located 777 km from Addis Ababa, Ethiopia and is the administrative centre of the Gambella Regional State. Gambella is characterized by having perennial malaria transmission due to uninterrupted suitable climatic conditions. Samples from this area were collected from Gambella hospital. West Arsi Zone is located 251 km from Addis Ababa, Ethiopia. Shashemene is the administrative centre of the West Arsi Zone. The zone has elevation ranging from 1,500 to 2,300 m above sea level. Malaria transmission is seasonal and unstable in the West Arsi Zone. Samples from this area were collected from Aje Health centre.

The samples in Tanzania were collected from Mkuzi in Muheza district, Tanga region, Nachingwea district in Lindi, Muleba district in Kagera region and Ilemela district in Mwanza region. These sites were selected to represent areas with different malaria transmission. The sites were selected based on the recent Tanzania HIV and malaria indicator survey whereby Nachingwea in Lindi have high malaria prevalence while Muleba in Kagera have moderate malaria transmission. Muleba is also one of the districts which are prone to malaria epidemics in the country. The sites, Muheza in Tanga and Ilemela in Mwanza have moderate malaria transmission. In all four sites, malaria is caused mainly by *P. falciparum*.

### Sample collection

Finger-prick blood samples were collected from patients attending clinics/hospitals in the above mentioned study sites in Ethiopia and Ilemela, Tanzania after obtaining patients’ informed consent. In the other three sites in mainland Tanzania, venous blood samples (3–5 ml) were collected and all blood samples were spotted on Whatman 3MM filter papers. The papers were first allowed to air-dry, the dried blood spots (DBS) were stored in self-sealing plastic bags for subsequent molecular analysis.

### Malaria diagnosis

Thick and thin blood smears stained with 10% Giemsa for 10 min in all three study sites in Ethiopia. The slides were read by two independent experienced lab technicians and malaria-infected patients were treated as per the national treatment guideline: AL for *P. falciparum*, CQ for vivax malaria. For the three study sites in Tanzania (Mkuzi, Nachingwea and Muleba), the slides were stained with 3% Giemsa for 45 min. Initial screening and treatment of patients were done using RDT and only a single microscopy reading was done as a confirmation. For determination of parasitaemia, infected red blood cells were counted in microscopic fields containing 200 leucocytes and then multiplied by 40 (assuming a standard mean white blood cell count of 8,000 leucocytes per μl of blood) [40]. A blood smear was considered to be negative if no parasite was seen after examining 200 fields.

### DNA extraction

*Plasmodium falciparum* DNA was extracted from DBS using the chelex extraction method as described elsewhere [41]. DNA obtained was transferred into 0.5 ml DNA tubes and kept in −20°C until use.

### Genotyping of the *pfcrt* gene

The *pfcrt* gene was analysed by PCR–RFLP using primers flanking residue 76 in two rounds of PCR with two pairs of outer and inner primers as described elsewhere [42]. The 145-bp nested PCR product was then digested with restriction enzyme *Xap*I (Thermo Scientific), which cleaves *pfcrt*-76K but not *pfcrt*-76T [42, 43]. All PCRs were run in a LifePro thermal cycler (Bioer Technology, Hangzhou, China).

### Genotyping of the *pfmdr*-1 gene

The *pfmdr*-1 was analysed as previously described elsewhere [5]. Parasite DNA was amplified with primers flanking codon 86 in two rounds of outer and nested PCR using two sets of primers. *Pfmdr*-1 alleles were then identified using *Apo*I (Thermo Scientific), which cleaves the coding sequence of allele *pfmdr*-1 86N but not that of *pfmdr*-1 86Y. The polymorphic asparagine rich linker region was amplified according to Duraisingh et al. [5] and PCR amplicons were analysed by nucleotide sequence determination at Uppsala Genome Centre. Sequencing reactions were run with AB BigDye Terminator v3.1 and spin-column based clean-up. Sequencing samples were separated by capillary electrophoresis on the ABI3730XL DNA Analyzer (Applied Biosystems).

### Genotyping of the *pfubp*-1 gene

Polymorphisms in *pfubp*-1 (PF3D7_0104300) in the 300 bp region encompassing codons 1463–1563 were determined using a previously published PCR strategy [33]. The products were characterized by direct sequencing.

### Statistical analysis

All statistical analyses were performed using STATA 13.0 (Texas, 77845 USA). The frequency of mutant and wild type alleles were calculated as the proportion of all the alleles at a given locus detected among all the parasite isolates examined. The 2-tailed Fisher’s exact test was used to compare all isolates from Ethiopia and Tanzania at the *pfcrt* K76T and *pfmdr*-1 N86Y alleles. Analysis of the *pfubp*-1 region was done by sequence comparison with the 3D7 wild type reference *P. falciparum* strain. Statistical significance was defined as a *P* value ≤0.05.

### Ethical considerations

Ethical approval for the study sites in Ethiopia was obtained from the Institutional Review Boards of Aklilu Lemma Institute of Pathobiology and Science Faculty, Addis Ababa University and the Armauer Hansen Research Institute as well as from the National Health Research Ethics Review Committee. For study in Mwanza, Tanzania, ethical clearance was obtained from Weill Bugando University College of Health Sciences and Bugando Medical Centre (WBUCHS/BMC) Ethical and Publication Committee. The study conducted at Mkuzi, Nachingwea and Muleba received ethical and scientific approval from the Medical Research Coordination Committee (MRCC) of the Tanzanian National Institute for Medical Research and the Ministry of Health and Social Welfare.

## Results

The prevalences of *pfcrt* 76T and *pfmdr*1 86Y mutations in three study sites in Ethiopia are shown in Table 1. Resistance to CQ was essentially fixed in two study sites in Ethiopia, West Arsi/Shalla and Adama, with 100% of the isolates harboring the *pfcrt* K76T mutation. In isolates from Gambella, the wild type alleles K76 and N86 of *pfcrt* and *pfmdr*-1 gene were present in 27.3% (6/22) and 73.9% (17/23) of the samples, respectively. The prevalence of *pfmdr*-1 86Y mutation was 2% in West Arsi, 23.3% in Adama and 26.1% in Gambella (Table 1).

**Table 1:** Frequencies of mutations in *pfcrt* 76 and *pfmdr*-1 86 codons in *P. falciparum* isolates from Ethiopia and Tanzania

Country/locations	*Pfcrt*			*Pfmdr*-1		
	76K, n (%)	76T, n (%)	Total	86N, n (%)	86Y, n (%)	Total
Ethiopia						
Adama	0 (0)	31 (100)	31	23 (76.7)	7 (23.3)	30
Gambella	6 (27.3)	16 (72.7)	22	17 (73.9)	6 (26.1)	23
West Arsi	0 (0)	99 (100)	99	49 (98)	1(2)	50
Tanzania						
Muleba	23 (85.2)	4 (14.8)	27	18 (81.8)	4 (18.2)	22
Nachingwea	31 (100)	0 (0)	31	27 (97.6)	0 (0)	27
Mwanza	20 (100)	0 (0)	20	7 (87.5)	1 (12.5)	8
Mkuzi	17 (85.0)	3 (15.0)	20	11 (55.0)	9 (45.0)	20

In Tanzania, the majority of the isolates carried wild type *pfcrt* 76K and *pfmdr*-1 86N genes. In two areas of Tanzania, Mwanza and Nachingwea, all isolates (100%) carried wild type *pfcrt* 76K gene (Table 1). The prevalence of *pfcrt* 76T mutation was 15 and 14.8% in Mkuzi and Muleba, respectively. The frequencies of wild type and mutant *pfmdr*-1 86 in the four study sites in Tanzania are shown in Table 1.

An overall comparison of isolates from Ethiopia and Tanzania at the *pfcrt* 76 and *pfmdr*-1 86 codons showed Tanzania to have a significantly higher prevalence of the wild type *pfcrt* 76K allele (91/98; 92.8%) compared to Ethiopia (6/152; 4.0%); p < 0.0001. However, no significant difference (p = 0.4138) was observed between isolates the two countries with regard to the *pfmdr*-1 86 alleles (63/77; 81.8%, and 89/103; 86.4% wild type in Tanzania and Ethiopia, respectively).

**Figure 1:**
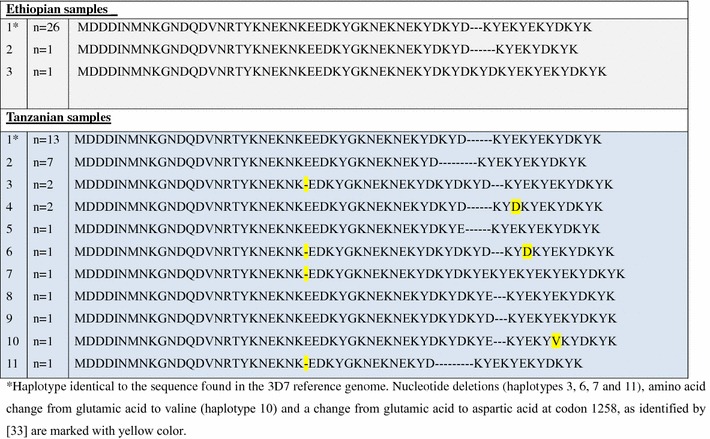
Haplotype frequencies at codons 1463–1563 of *pfubp*-1. *Haplotype identical to the sequence found in the 3D7 reference genome. Nucleotide deletions (haplotypes 3, 6, 7 and 11), amino acid change from glutamic acid to valine (haplotype 10) and a change from glutamic acid to aspartic acid at codon 1258, as identified by [33] are marked with *yellow color*.

**Figure 2:**
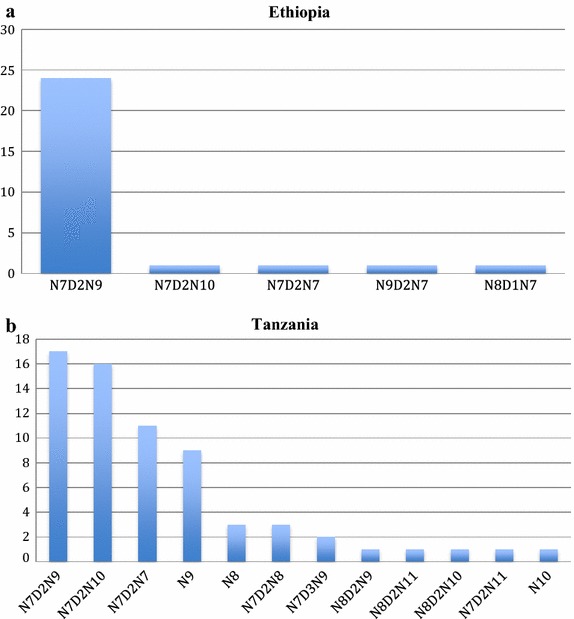
Analysis of variation in linker region of *pfmdr*-1gene among isolates from Ethiopia (**a**) (n = 28) and Tanzania (**b**) (n = 66) samples.

Direct sequencing of *pfubp*-1gene was possible for 42 Tanzanian and 28 Ethiopian samples (Figure 1). Three different types of haplotypes were identified among the Ethiopian isolates (one haplotype identical to 3D7, and 2 other variants). Of 28 Ethiopian isolates, 26 isolates (92.8%) had identical genome sequences with 3D7 reference genome sequence (Figure 2a). There were 11 different types of genome sequences identified among the Tanzanian isolates. Of 42 isolates successfully analysed from Tanzania, only 13 (30.9%) had identical genome sequences with 3D7 (Figure 1). In the Tanzanian samples, several new variants were found in comparison with the earlier published sequence variation [34]. The second most common variant, haplotype 2, had a deletion of three amino acids compared to the 3D7 reference sequence. Haplotypes 3, 6, 7 and 11 had deletion of one glutamic acid, in a part where [34] found insertions of one glutamic acid in several isolates. Haplotype 11 had in addition the same three amino acid deletion as haplotype 2. Finally, haplotype 10 showed a single amino acid change from glutamic acid to valine. Haplotypes 4 and 6 showed a change from glutamic acid to aspartic acid at the position indicated earlier [33].

Analysis of the sequence variation in *pfmdr*-*1* variable region was possible for a total of 13 isolates from Ethiopia and 50 from Tanzania (Figure 2). The variable linker region in *pfmdr*-1 consists of consecutive NDN residues. Except for four isolates from Gambella, all isolates from Ethiopia had one haplotype sequence, N7D2N9 (Figure 2a) while the Tanzanian samples had twelve different haplotypes (Figure 2b). The dominating variants in Tanzania were N7D2N9 and N7D2N10 with almost equal frequencies. Representative sequences for all variants were deposited at the European Nucleotide Archive with accession numbers LN794745-LN794770 (http://www.ebi.ac.uk/ena/data/view/LN794745-LN794770).

## Discussion

Genotyping of *pfcrt* and *pfmdr*-1 markers in Ethiopia showed distinct frequencies of wild type and mutant parasite population. The *pfcrt* 76T mutation was 100% in isolates collected from West Arsi Zone and Adama, central Ethiopia. The wild type *pfcrt* 76K was identified in 27.3% of the isolates from Gambella, southwestern Ethiopia (*pfmdr*-1 86N). For *pfmdr*-186 codon the majority of the parasites from three study sites in Ethiopia were wild type. Moreover, analysis of the variable linker region in *pfmdr*-1 showed the presence of one haplotype. Analysis of the *pfubp*-1 region in isolates from Ethiopia showed no divergence from the *P. falciparum* 3D7 reference strain, but large variations from 3D7 in Tanzanian isolates.

In the samples collected from two sites in Ethiopia (West Arsi and Adama), it has been reported that the K76T mutation was present in all isolates examined while the wild type amino acid (K) was found in 27.3% of samples analysed from Gambella. In all the three study sites in Ethiopia, the wild type *pfmdr*-1 86N dominated the parasite population. The variation in the frequencies of mutant and wild type in the *pfcrt* 76 and *pfmdr*-1 86 codons could be due to the differential selection of the isolates by the current regimen used for the treatment of vivax and falciparum malaria in Ethiopia, CQ and AL, respectively. The selection of *pfmdr*-1 86N by AL has been shown in a number of studies [1, 8–10, 44]. Under CQ pressure, *pfcrt* 76T alleles spread more rapidly and reached high frequencies faster than *pfmdr*-*1* 86Y alleles, suggesting that CQ selection acts more strongly on *pfcrt* than on *pfmdr*-1 [44]. The high prevalence of the CQ sensitive *pfmdr*-1 86N allele signals the survival advantage of the wild type over its counterpart mutant forms in the currently used AL for falciparum malaria in Ethiopia as has been shown the selection of this allele by AL elsewhere [9].

Unlike in Ethiopia, the majority of the isolates collected from the four study sites in Tanzania carried the wild type alleles for both *pfcrt* and *pfmdr*-1. The frequencies of mutant and wild type alleles at *pfcrt* and *pfmdr*-1 loci not only exhibited variation between samples in the two countries but also variation among samples within the country. The observed variation in the frequencies of mutant and wild type variants for *pfcrt* and *pfmdr*-1 loci in the three study sites in Ethiopia could likely be explained by variation in transmission intensities and drug pressure *per se* in each study area. A study in the southern and eastern parts of Ethiopia by [45] showed the return of wild type alleles for both the *pfcrt* 76 and *pfmdr*-1 codons. In support of the present findings that showed variation in frequencies of mutant and wild type variants at *pfcrt* locus in three study sites in Ethiopia, the patchy distribution of *pfcrt* 76T resistant alleles was also seen in Laos, Southeast Asia [46]. Variation in *pfmdr*-1 and *pfcrt* genes among *P. falciparum* populations in different regions in Sudan, Tanzania and Uganda, and likely reflects variation in drug pressure between each region [47, 48]. Even under prolonged drug pressure that favors resistant parasites to overwhelm, in countries where transmission is high and acquired immunity is extensive, asymptomatic adults who are less susceptible and who generally do not receive therapy, may provide a reservoir for susceptible parasites to persist in the population that may regain predominance when the pressure is removed [14].

The persistence for selection of the mutant genotype, *pfcrt* 76T, in Ethiopia could be attributed to the absence of complete CQ withdrawal because studies have shown that in areas where drug usage has decreased, the spread of resistance has also decreased [15, 49]. To completely replace the less-fit CQ resistance genotype, complete withdrawal of the drug is required [50]. In Uganda, for instance, where CQ withdrawal was incomplete, about 8 years post-CQ replacement *pfcrt*76T prevalence was between 100 and 98.7% [37, 51]. In Ethiopia, the at-risk population continues to inadvertently expose *P. falciparum* to CQ pressure due to CQ-based *P. vivax* treatment in the country. Within 8 years of CQ withdrawal in Malawi [15], for instance, the prevalence of the chloroquine-resistant *pfcrt* 76T genotype decreased from 85% in 1992 to 13% in 2000. In 2001, CQ cleared 100% of 63 asymptomatic *P. falciparum* infections, no isolates were resistant to CQ in vitro, and no infections with the CQ-resistant *pfcrt*76K allele were detected. In Cambodia and Thailand, CQ remains the first-line treatment for *P. vivax* malaria and continues to select for the resistant genotype in *P. falciparum* leading to the fixation of *pfcrt*-K76T alleles [52].

Beyond the *pfcrt* K76T and *pfmdr*-1 N86Y alleles, a substantial sequence variation in *pfmdr*-*1* variable region was noted in isolates from Ethiopia and Tanzania. The low variability in the polymorphic region of *pfmdr*-*1* in Ethiopia may be a consequence of low transmission intensity in the country compared to high transmission intensity in Tanzania. However, it should be noted that the majority of the Tanzanian isolates, just like the Ethiopian parasites, carried the wild type *pfmdr*-1 N86 genotype. The similar prevalence of the *pfmdr*-1 86N allele in both countries could be reflective of its selection by AL pressure, since the AL is currently the first-line therapy for uncomplicated *P. falciparum* malaria in both countries.

The polymorphism at *pfubp*-1 codon 1528 previously identified in Kenyan samples [33], was not detected in Ethiopian samples but was found in two Tanzanian isolates. Overall, nucleotide variability was low in Ethiopia and most isolates conformed to the 3D7 reference sequence. The variability in Tanzania was strikingly larger, and included several new haplotypes not reported in an earlier publication [34]. However, because all samples used in the present study were from patients who had been successfully treated with ACT and followed only for 3 days after treatment (except samples collected from Nachingwea) with no clear signs of slower clearance, it is difficult to make any conclusions with regard the possible involvement of this *pfubp*-1 1528 SNP or the new haplotypes in AL response in the study areas under report. Further studies with longer follow up would be needed to correlate variation in *pfubp*-*1* with increasing risk for recrudescence/reinfection.

The *pfcrt* and *pfmdr*-1 allele frequencies reported here will be useful in the monitoring of rate of reversal of resistant forms of these alleles following withdrawal of CQ use as a first-line treatment in Ethiopia and Tanzania.

## Conclusion

In conclusion, *pfcrt* 76T mutation was high in Ethiopia several years after the change in drug policy unlike other studies wherein decline in drug- resistance-associated mutations or resistant parasites have been observed. Even though CQ is no longer prescribed for uncomplicated *P. falciparum* malaria in Ethiopia, it continues to be the standard of care for *P. vivax*, and this may have led to fixation of the CQ-resistant genotype in the study sites in Ethiopia. On the other hand, the prevalence of mutant *pfmdr*-1 allele decreased in these samples. In Tanzania, a decreased prevalence of *pfcrt* and *pfmdr*-1 mutations was observed. To better understand the patterns of recovery of CQ susceptibility, it is recommend further molecular epidemiological studies in different endemic areas having different histories of CQ usage. The AL sensitivities of isolates with different haplotypes residues in Ethiopia and Tanzania need to be determined.
